# Mechanical Behaviors of Angle-Ply Black Phosphorus by Molecular Dynamics Simulations

**DOI:** 10.3390/nano8100758

**Published:** 2018-09-26

**Authors:** Lili Li, Rui Sun, Jie Yang

**Affiliations:** School of Engineering, RMIT University, PO Box 71, Bundoora, VIC 3083, Australia; lili.li@rmit.edu.au (L.L.); s3582253@student.rmit.edu.au (R.S.)

**Keywords:** double-layer black phosphorus, angle-ply microstructure, stacking angle difference, mechanical behaviors, molecular dynamics simulation

## Abstract

Regular black phosphorus (BP) sheets possess strongly anisotropic properties due to the unique puckered atomistic configuration, making such BP mechanically very weak in the armchair direction. The present work aims to address this issue by proposing an angle-ply double-layer black phosphorus (DLBP) structure in which two individual atomic layers with different orientation angles are stacked up. The molecular dynamics simulations based on Stillinger-Weber potential show that the in-plane mechanical properties of such a DLBP structure, e.g., Young’s modulus and tensile strength are significantly influenced by the stacking angle of each layer. The property anisotropy of DLBP decreases as the stacking angle difference *δ* between two layers increases and becomes isotropic when *δ* = 90°. This work also shed insight into mechanisms of angle-ply layers underlying the mechanical behaviors of DLBP at the nanoscale, suggesting that the anisotropic material properties can be effectively controlled and tuned through the appropriately selected stacking angles.

## 1. Introduction

As a new two-dimensional (2D) layered nanomaterial reintroduced recently, black phosphorus (BP) has immediately drawn extensive interests due to its exceptional anisotropic electrical and optical properties [1,2,3,4,5,6,7,8,9,10]. Moreover, BP possesses a tuneable direct band gap that can be tuned in a wide range from around 0.3 eV to 2.0 eV depending on its number of atomic layers [11,12] and in-plane mechanical strain [13,14]. The excellent combination of these peculiar properties makes BP a promising material for novel applications in electrical and optical fields [3,4,5,6,7,8,9,10,11,12,15,16,17] that is different from graphene and transition metal dichalcogenides (TMDs), such as tunable photodetection accessing a wide spectrum ranging from visible to infrared regimes [3,4,5,6], semiconductors, and alternative electronic materials to graphene and MoS_2_ for transistor applications [6,7,8,9,10,15,16,17], heterostructures [18,19], and solar cells [20,21].

Various fabrication techniques have been reported [22,23] to produce BP flakes with considerably improved chemical stability and significantly reduced degradation. Although extensive experimental and numerical studies on BP have been conducted, most of them are focused on the electrical and optical properties while research work on its mechanical behavior is rather limited. For layered anisotropic materials, various analytical and experimental methods have been employed to investigate their mechanical properties, such as indentation tests [24,25], molecular dynamics (MD) simulations [26,27,28,29,30], and first-principle calculations [12].

It has been shown in previous investigations that BP is strongly anisotropic due to its puckered atomic configuration. For example, its Young’s modulus in the zigzag direction (~58.6–159 GPa) is ca. four times more than that in the armchair direction (~19.5–41.3 GPa) [5,12,26,27,28,29,30]. Jiang et al. [31] found that BP’s Poisson’s ratio is also anisotropic and reported an apparent negative Poisson’s ratio in a single-layer black phosphorus (SLBP) when it is subjected to a uniaxial in-plane stress in the pucker (zigzag) direction and observed a positive Poisson’s ratio when the SLBP is under the stress in the perpendicular (armchair) direction. It is also demonstrated in our previous works [29,30] that the application of an armchair-oriented compressive prestrain can improve BP’s mechanical properties much more significantly than a zigzag-oriented prestrain does. Since the inherent anisotropic material properties make a mechanically loaded BP much weaker and more vulnerable to failure in the armchair direction, it is of crucial importance to reduce the anisotropy to achieve balanced properties in both directions for engineering applications.

To this end, this paper proposes a modified atomic structure named double-layer black phosphorus (DLBP) by stacking up two single BP layers with different orientation angles to form an angle-ply structure whose mechanical properties and anisotropy can be tuned by changing the stacking angle difference between the two layers. The atomic configurations of the angle-ply DLBP with various stacking angles are constructed and their tensile behaviors are investigated by using MD simulations. The relationship between the stacking angle difference and material properties is discussed in detail and the condition for the DLBP to become an isotropic BP is identified. To the best of the authors’ knowledge, no previous work has been done to address this important issue.

## 2. Methodology

**Figure 1:**
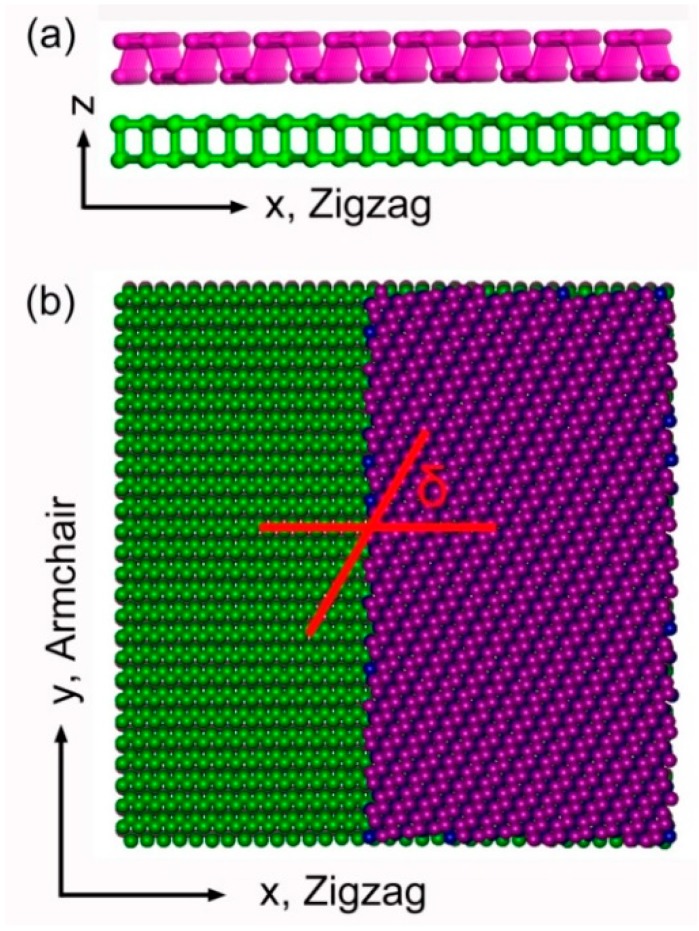
Atomic structure of double-layer black phosphorus (DLBP) crystal with a stacking angle difference *δ* = 60° from (**a**) side view and (**b**) top view. The atoms colored by green and purple are in the bottom and top layer, respectively. For a better view, only half of the top layer is shown in (**b**).

The atomic structure of the proposed angle-ply DLBP is shown in Figure 1 where zigzag (X-) and armchair (Y-) directions of the bottom layer are parallel and perpendicular to the puckers, respectively. The dimension of the unit cell is 103 Å × 103 Å. To create such an angle-ply microstructure, the regular DLBP is constructed first then the bottom atomic layer is fixed in its original position while the top layer is rotated along clockwise direction by a certain angle to form a DLBP with stacking angle difference *δ* = *θ*_T_ − *θ*_B_, where *θ*_T_ and *θ*_B_ denote the stacking angle of the top and bottom layers, respectively.

In MD simulations, the entire system is fully relaxed to equilibrium state as the first step. The tension is applied through the following steps: (1) all atoms at one end of the DLBP are held fixed in the loading direction and remain unconstrained in the out-of-plane direction; (2) apply displacement to all atoms at the other end at a constant strain rate of 10^−4^ ps^−1^, while all other atoms are free to relax in time. Free boundary conditions are applied in three dimensions.

The MD algorithm used here is implanted in the LAMMPS (2016) code [32] to simulate the uniaxial tension of the angle-ply DLBP. During simulations, the time step of 1 fs is used to integrate the atomic motion equation. The forces among P atoms in each layer of DLBP are calculated by Stillinger-Weber potential recently developed for SLBP [26,27]. This potential is parameterized based on the valence force field and the computed phonon spectrum and the mechanical properties of BP thus obtained agree quite well with ab initio calculations [27]. The atom-atom interactions between two layers are described by the Lennard-Jones potential parameterized for bilayer BP [33], which is proven to be very accurate for computing the interlayer cohesive energy and frictional energy for layered BP [33,34]. The thickness of the single layer is taken as 5.24 Å [27].

## 3. Results and Discussion

**Figure 2:**
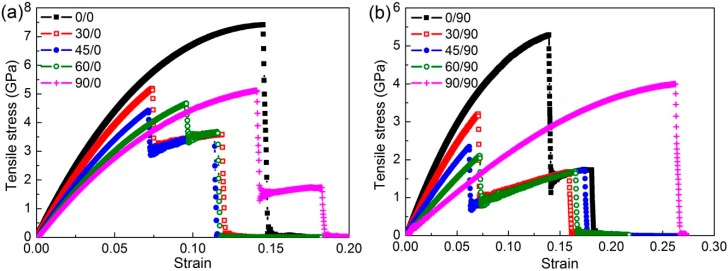
Tensile stress-strain curves of the angle-ply DLBP in (**a**) X-axis; and (**b**) Y-axis directions. The numerical numbers in legend denote stacking angle *θ*_T_/*θ*_B_.

**Figure 3:**
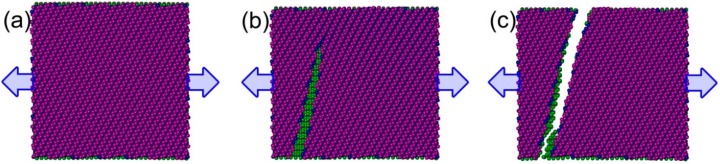
Top view of the atomic configurations of the angle-ply DLBP with *δ* = 60° at selected stages during tensile process in the X-axis: (**a**) *ε* = 0; (**b**) *ε* = 0.096; and (**c**) *ε* = 0.117. The stacking angles *θ*_T_ = 60° and *θ*_B_ = 0°.

Figure 2 shows the tensile stress-strain curves in two in-plane directions for DLBP with various stacking angle differences. As can be observed, the tensile stress increases almost linearly as strain increases up to a critical value (i.e., the tensile strength), followed by an abrupt drop. It is interesting to note that the stress of the regular DLBP, whose stacking angle difference *δ* = 0 drops to zero, showing a typical brittle fracture mode which is also observed in other nanomaterials [35]. However, the stress exhibits a two-step drop when *δ* ≠ 0, indicating that the two atomic layers in the angle-ply DLBP break at two separate stages. This can be clearly interpreted by examining its deformed atomic configuration at failure shown in Figure 3 where the snapshots of DLBP’s configuration with *δ* = 60° at selected stages in the tension process are given. It is seen that the top layer breaks first at strain *ε* = 0.096 (corresponding to the first drop of the curve *θ*_T_/*θ*_B_ = 60°/0° in Figure 2a) while the bottom layer remains intact and capable of sustaining further strain until the entire system eventually fails at the ultimate strain *ε* = 0.117 (represented by the lower section of the stress-strain curve with 60°/0° that ends up with a steep drop to zero in Figure 2a). This is because the regular DLBP with *δ* = 0 can accommodate more external strain through the change in the pucker/zigzag angle instead of the bond length when it is stretched [28,29,30,34]. However, the rotated top layer in the angle-ply DLBP limits the capability of the pucker/zigzag angle change and, as a result, breaks earlier than the bottom layer.

It is also worthy to note from Figure 3b that the crack angle along which the crack grows in the rotated top layer is seen to be larger than the stacking angle *θ*_T_ = 60°. This deviation is primarily owing to the “strengthening” effect induced from the bottom layer which has not been damaged yet. It should be mentioned that this phenomenon also occurs in other angle-ply DLBP considered in this work, except those with *δ* = 90°. Meanwhile, the fractured top layer weakens the DLBP nanostructure, hence both tensile strength and ultimate strain become lower than those of the regular DLBP (*δ* = 0°) except the case *δ* = 90°, as shown in Figure 2a.

It is interesting to note that for the angle-ply DLBPs with *δ* = 90° that are denoted by 90°/0° in Figure 2a and 0°/90° in Figure 2b, respectively, the stress-strain curves in both X- and Y-axes are almost identical. This is due to the fact that 90°/0° and 0°/90° are two special cases where the armchair (or zigzag) directions of the top and bottom layers are perpendicular to each other. Therefore, the angle-ply DLBP with stacking angles of *θ*_T_/*θ*_B_ with 90°/0° under a uniform tension in the X- (Y-) direction exhibits the same behavior as the DLBP with 0°/90° being uniformly stretched in the Y- (X-) direction. During the tensile process, the DLBP is elongated uniformly up to the critical strain where the zigzag layer (i.e., the zigzag orientation is parallel to the loading direction) fails, followed by the failure of the armchair layer (i.e., the armchair orientation is parallel to the loading direction) at its ultimate strain. This indicates that the armchair layer contributes lower strength, but higher ultimate strain in the DLBP with *δ* = 90° (90°/0° in Figure 2a), than in the regular zigzag DLBP (0°/0° in Figure 2a), while the zigzag layer contributes higher strength but lower ultimate strain in the DLBP with *δ* = 90° (0°/90° in Figure 2b) than in the armchair DLBP (90°/90° in Figure 2b).

**Figure 4:**
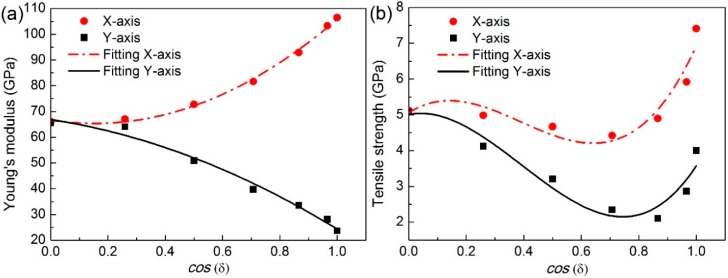
The effect of stacking angle difference *δ* on (**a**) Young’s modulus, and (**b**) tensile strength of the angle-ply DLBP during the tension process along both X- and Y-axes.

From the stress-strain curves, Young’s modulus is determined from the slope of the linear region (strain *ε* ≤ 0.01) in the curve using linear regression, while the tensile strength is defined as the tensile stress corresponding to the critical strain at the first drop in the curve. Figure 4 depicts the effect of stacking angle difference *δ* on DLBP’s Young’s modulus and tensile strength in which cos(*δ*) = 1 (i.e., *δ* = 0°) corresponds to a regular DLBP consisting of two identical BP layers whose Young’s modulus and tensile strength in the X-axis (zigzag direction) are much higher than those in the Y-axis (armchair direction) due to the anisotropic lattice [26,27,28,29,30,34]. It can be seen from Figure 4a that as the value of cos(*δ*) decreases (i.e., *δ* increases), Young’s modulus becomes lower in the X-axis but higher in the Y-axis. In other words, the DLBP tends to be less anisotropic as the zigzag orientation of the top layer deviates from the X-axis towards the Y-axis which is the armchair direction of the bottom layer. This can be explained by the fact that the regular DLBP with *δ* = 0° has the zigzag orientations of both layers in the X-axis and the armchair orientations in the Y-axis is, hence, the strongest in the X-axis and the weakest in the Y-axis. Therefore, the derivation of the top layer weakens the DLBP in the X-axis, but at the same time strengthens it in the Y-axis, making the material properties in the X- and Y-axes become closer and closer as the stacking angle difference *δ* increases, and eventually the angle-ply DLBP turns to be isotropic at *δ* = 90°.

In addition to Young’s modulus, DLBP’s tensile strength is also strongly influenced by the stacking angle difference *δ*, as shown in Figure 4b. The tensile strength of the regular DLBP with the value of cos(*δ*) = 1 (i.e., *δ* = 0) is found to be ~7.4 GPa in the X-axis (zigzag direction) and ~4.0 GPa in the Y-axis (armchair direction), showing a remarkable anisotropy. These values agree quite well with those reported in previous studies [26,27,28,29,30,34]. As *δ* increases, the anisotropy becomes weaker and finally disappears at cos(*δ*) = 0 (i.e., *δ* = 90°). This is consistent with the results for Young’s modulus. It should be noted that, unlike Young’s modulus, the tensile strength varies non-monotonically with increasing values of cos(*δ*). This is attributed to the combined influence of both the bond type (i.e., the puckered bond or the zigzag one) and the stacking angle difference *δ*.

Using the least squares fitting method, the relationship between Young’s moduli *E*, tensile strengths *Y* in both X- and Y- axes, and cos(*δ*) can be obtained as:

(1) $$E_{\text{X-axis}}=56.158\cdot \cos^{2}(\delta )-16.988\cdot \cos(\delta )+66.640$$

(2) $$E_{\text{Y-axis}}=-25.489\cdot \cos^{2}(\delta )-17.044\cdot \cos(\delta )+66.867$$

(3) $$\sigma_{\text{X-axis}}=19.009\cdot \cos^{3}(\delta )-22.295\cdot \cos^{2}(\delta )+5.153\cdot \cos(\delta )+5.058$$

(4) $$\sigma_{\text{Y-axis}}=16.611\cdot \cos^{3}(\delta )-19.559\cdot \cos^{2}(\delta )+1.514\cdot \cos(\delta )+5.011$$

In other words, Equations (1)–(4) can be used to predict the mechanical properties of such two-dimensional angle-ply BP structure.

**Figure 5:**
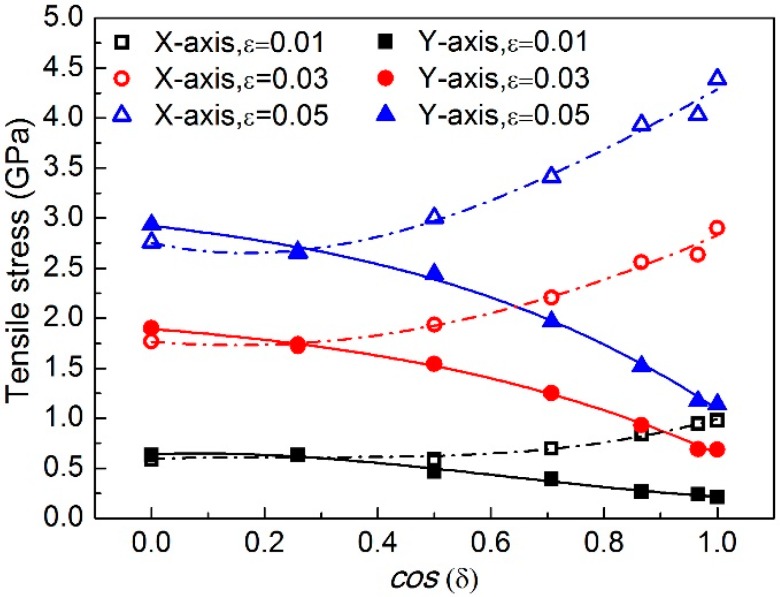
Evolution of tensile stress at selected strain levels during the loading with respect to the stacking angle difference *δ*.

Moreover, the relationship between tensile stress and the stacking angle difference *δ* at selected strain levels during the loading is investigated, as shown in Figure 5. Interestingly, the evolution of stress with respect to *δ* exhibits similar characteristic with that of Young’s modulus shown in Figure 4, i.e., both tensile stress and Young’s modulus present as polynomial at the order of the square to the cosine of *δ*.

Using the least squares fitting method, the relationship between tensile stress *σ* in both X- and Y- axes and cos(*δ*) can be obtained as:

(5) $$\sigma_{\text{X-axis}}=50.731\cdot \varepsilon \cdot \cos^{2}(\delta )-16.568\cdot \varepsilon \cdot \cos(\delta )+59.771\cdot \varepsilon$$

(6) $$\sigma_{\text{Y-axis}}=-30.586\cdot \varepsilon \cdot \cos^{2}(\delta )-10254\cdot \varepsilon \cdot \cos(\delta )+58.672\cdot \varepsilon$$

In other words, Equations (5) and (6) can be used to predict the tensile stress of such angle-ply DLBP at various strain levels.

## 4. Conclusions

An angle-ply DLBP has been proposed and its tensile behaviors have been investigated by employing MD simulations. Results show that DLBP’s mechanical properties and anisotropy are highly dependent on the stacking angle difference *δ*. As *δ* increases, both Young’s modulus and tensile strengths in the X- and Y- directions tend to be closer and the nanostructure becomes less anisotropic. The anisotropy eventually vanishes and the DLBP turns to be isotropic at *δ* = 90°. The present study suggests that BP’s material properties and its inherent anisotropy can be effectively controlled and tuned by the proposed angle-ply DLBP nanostructure to achieve the desired mechanical performances.
